# Repressed Ang 1–7 in COVID-19 Is Inversely Associated with Inflammation and Coagulation

**DOI:** 10.1128/msphere.00220-22

**Published:** 2022-08-01

**Authors:** Rebecca M. Carpenter, Mary K. Young, William A. O. Petri, Genevieve R. Lyons, Carol Gilchrist, Robert M. Carey, William A. Petri

**Affiliations:** a Division of Infectious Diseases and International Health, Department of Medicine, University of Virginia School of Medicine, Charlottesville, Virginia, USA; b Division of Endocrinology and Metabolism, Department of Medicine, University of Virginia School of Medicine, Charlottesville, Virginia, USA; c Integrated Translational Health Research Institute (iTHRIV), University of Virginia School of Medicine, Charlottesville, Virginia, USA; d Department of Microbiology, Immunology and Cancer Biology, University of Virginia School of Medicine, Charlottesville, Virginia, USA; e Department of Pathology University of Virginia School of Medicine, Charlottesville, Virginia, USA; Mount Sinai School of Medicine

**Keywords:** Ang 1–7, COVID-19, coagulation, inflammation, renin-angiotensin system

## Abstract

The coronavirus SARS-CoV-2 infects host cells by binding to the angiotensin-converting enzyme 2 (ACE2) receptor, which belongs to an anti-inflammatory, anti-thrombotic counter-regulatory arm of the renin-angiotensin system (RAS). ACE2 dysfunction and RAS dysregulation has been explored as a driving force in acute respiratory distress syndrome (ARDS), but data from COVID-19 patients has been inconsistent and inconclusive. We sought to identify disruptions of the classical (ACE)/angiotensin (Ang) II/Ang II type-1 receptor (AT_1_R) and the counter-regulatory ACE2/Ang 1-7/*Mas* Receptor (*Mas*R) pathways in patients with COVID-19 and correlate these with severity of infection and markers of inflammation and coagulation. Ang II and Ang 1–7 levels in plasma were measured by enzyme-linked immunosorbent assay (ELISA) for 230 patients, 166 of whom were SARS-CoV-2+. Ang 1–7 was repressed in COVID-19 patients compared to that in SARS-CoV-2 negative outpatient controls. Since the control cohort was less sick than the SARS-CoV-2+ group, this association between decreased Ang 1–7 and COVID-19 cannot be attributed to COVID-19 specifically as opposed to critical illness more generally. Multivariable logistic regression analyses demonstrated that every 10-pg/mL increase in plasma Ang 1–7 was associated with a 3% reduction in the odds of hospitalization (adjusted odds ratio [AOR] 0.97, confidence interval [CI] 0.95 to 0.99) and a 3% reduction in odds of requiring oxygen supplementation (AOR 0.97, CI 0.95 to 0.99) and/or ventilation (AOR 0.97, CI 0.94 to 0.99). Ang 1–7 was also inversely associated with pro-inflammatory cytokines and d-dimer in this patient cohort, suggesting that reduced activity in this protective counter-regulatory arm of the RAS contributes to the hyper-immune response and diffuse coagulation activation documented in COVID-19.

**IMPORTANCE** Severe acute respiratory syndrome coronavirus 2 (SARS-CoV-2) causes a unique disease, COVID-19, which ranges in severity from asymptomatic to causing severe respiratory failure and death. Viral transmission throughout the world continues at a high rate despite the development and widespread use of effective vaccines. For those patients who contract COVID-19 and become severely ill, few therapeutic options have been shown to provide benefits and mortality rates are high. Additionally, the pathophysiology underlying COVID-19 disease presentation, progression, and severity is incompletely understood. The significance of our research is in confirming the role of renin-angiotensin system dysfunction in COVID-19 pathogenesis in a large cohort of patients with diverse disease severity and outcomes. Additionally, to our knowledge, this is the first study to pair angiotensin peptide levels with inflammatory and thrombotic markers. These data support the role of ongoing clinical trials examining renin-angiotensin system-targeted therapeutics for the treatment of COVID-19.

## INTRODUCTION

Since its discovery in December 2019, SARS-CoV-2 has caused millions of cases of COVID-19 with highly variable symptoms, including but not limited to fever, cough, shortness of breath, fatigue, new loss of taste or smell, and diarrhea (1, 2). Clinical responses range from minor symptoms to hyperimmune activation, hypercoagulopathy, multiorgan dysfunction, and respiratory failure, leading to prolonged ICU admissions and death (2, 3). Two years into the pandemic, ongoing worldwide transmission even among vaccinated populations highlights the urgent need for further characterization of the pathophysiology of this disease and the continued development of effective therapeutics (1).

**FIG 1:**
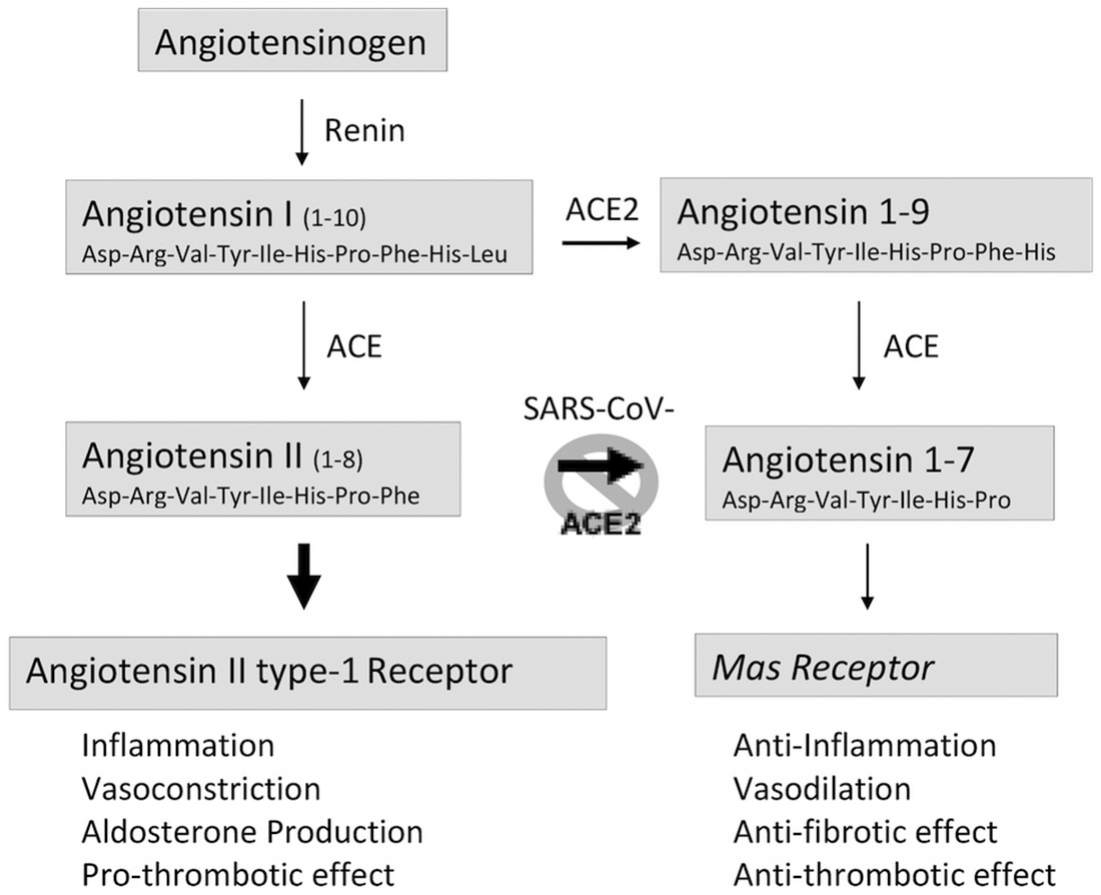
Schematic representation of the systemic renin-angiotensin system. Renin converts angiotensinogen into angiotensin I (Ang I). Angiotensin-converting enzyme (ACE) converts Ang I into angiotensin II (Ang II), which binds to Ang II type 1 receptor (AT_1_R), through which it exerts its harmful inflammatory effects. Angiotensin-converting enzyme 2 (ACE2) converts the majority of Ang II to Ang 1–7, which activates the *Mas* receptor (*Mas*R) signaling pathway with protective downstream effects on the microcirculatory environment. Attachment of SARS-CoV-2 spike protein to ACE2 induces cleavage and release of the soluble form of ACE2 by ADAM-17, virus-receptor complex internalization and receptor downregulation. Reduced ACE2 expression is hypothesized to cause an imbalance between the classical ACE/Ang I/AT_1_R and the protective ACE2/Ang 1–7/*Mas*R axes that is central to the pathophysiology of coronavirus disease 2019 (COVID-19).

The SARS-CoV-2 virus infects hosts by gaining entry to cells via its receptor angiotensin-converting enzyme 2 (ACE2), which belongs to the renin-angiotensin system (RAS), a cascade of biologically active peptides, enzymes, and receptors central to fluid and electrolyte balance and the regulation of blood pressure (4). Over the past 2 decades, ACE2 and its heptapeptide product angiotensin Ang 1–7 have increasingly been recognized as counterregulatory modulators of the classical RAS via activation of the *Mas* receptor (*Mas*R) (5). ACE2 regulates the RAS by converting Ang I to Ang 1–9 and cleaving a single amino acid from Ang II to form Ang 1–7 which is 500-fold more catalytically active than any other pathway leading to the formation of Ang 1–7 (6). Ang 1–7 then exerts its biological effects by activating the *Mas*R (Fig. 1) (7). Ang II acts via the Ang II type-1 receptor (AT_1_R) to induce vasoconstriction, inflammatory cytokine production, and extracellular matrix synthesis (8). Ang II also stimulates adrenal aldosterone production, leading to sodium and fluid retention and an increase in blood pressure (9). In contrast, Ang 1–7, via the *Mas*R, induces vasodilation and inhibits the production of proinflammatory cytokines along with other effects opposing those of Ang II (Fig. 1) (9).

RAS dysregulation has been hypothesized to have a central role in the pathogenesis of severe coronaviral infections as early as 2003, after the first SARS-CoV outbreak (8). After identification of ACE2 as the viral spike protein binding site, evidence emerged that ACE2 downregulation provides a molecular explanation for the observed severe respiratory failure caused by this virus (10). Multiple studies in animals and humans have shown that attachment of the viral spike protein to the ACE2 receptor at least transiently reduces ACE2 expression by several mechanisms: induced cleavage and release of the soluble form of ACE2 by ADAM-17, virus-receptor complex internalization, and receptor downregulation as a host-defense mechanism (10–12). *In vitro* and *in vivo* animal studies further established evidence that ACE2 is an important modulator of acute lung injury, including the most severe form, acute respiratory distress syndrome (ARDS) (5, 10, 13–16). Indeed, administration of the SARS-CoV spike protein in an acute lung injury mouse model reduces ACE2 expression and worsens the severity of injury (10). ACE2 knockout mice are shown to have worsened oxygenation, increased inflammation, and lung edema in ARDS induced by acid aspiration or sepsis (16). In terms of disease pathogenesis, ACE2 is thought to have a protective role in lung injury and to act in opposition to ACE by downregulating and thereby mitigating the pro-inflammatory effects of Ang II and promoting the effects of Ang 1–7 via the *Mas* receptor (Fig. 1) (15–18). In support of this hypothesis, the administration of recombinant ACE2 has been shown to reduce Ang II, increase Ang 1–7, and attenuate lung injury in acid-treated mice (16). These data support a robust hypothesis that COVID-19 is an acquired molecular disease characterized by RAS dysregulation, namely, ACE2 downregulation, which leads to an exacerbated immune response, microcirculatory thrombosis, and fibrosis (18, 19).

Observations from animal models have suggested RAS modulation as a potential therapeutic mechanism to treat COVID-19, and clinical trials examining the impact of Ang 1–7 and human recombinant ACE2 are ongoing. However, evidence from human data has shown that these hypotheses have perhaps been too optimistic (8). Early studies have shown conflicting results, with evidence pointing toward overactivation of the ACE2/Ang 1–7/MasR pathway and an overall increase in Ang 1–7 in COVID-19 (8). Furthermore, little to no data in human studies supports the role of RAS dysregulation leading to the hypothesized downstream inflammatory and thrombotic overactivation in COVID-19. Thus, there is a need for further investigation to determine whether RAS dysfunction is responsible for at least some of the unique features of COVID-19 and to support the development of targeted therapeutics. Here, we measured Ang II and Ang 1–7 as proxies for ACE2 activity in the plasma of COVID-19 patients to determine their relationship to illness severity and inflammation and coagulopathy markers.

## RESULTS

### Patient characteristics.

The clinical and demographic characteristics of COVID-19 patients and control subjects are displayed in Table 1. The average age of COVID-19 patients was 57.1 ± 17.7 years (14 to 104 min-max), and 55% were males. The average age of control subjects was 51.6 ± 20.2 years (9 to 90 min-max), and there was no significant difference in gender distribution compared to the SARS-CoV-2 positive group. A significantly lower percentage of COVID-19 patients compared to control subjects was Caucasian and a significantly greater percentage was Hispanic. Significant differences were noted in body mass index (BMI) and cancer and diabetes diagnoses between the SARS-CoV-2-positive and -negative groups. The majority of patients had at least one comorbidity. Among the 166 COVID-19 patients, 142 were hospitalized, 118 required oxygen supplementation, 61 required ventilation, and 25 died of the disease. Demographics and characteristics by patient outcomes are shown in Table S1 in the supplemental material. On average, the samples used for this study were collected 10.5 days after symptom onset. In total, 73 COVID-19 patients (44.0%) received steroids and 39 (23.8%) received Remdesivir. Of note, while ACE inhibitor (ACEi) and angiotensin receptor blocker (ARB) use were included in this analysis, these prescribed medications represent long-term use and were not administered during the acute hospitalization.

**TABLE 1:** Patient demographics and clinical characteristics stratified by COVID-19 (*n* = 230)^*a*^

Characteristic	COVID− (*n* = 64)^*b*^	COVID+ (*n* = 166)^*b*^	*P* value^*c*^
Demographics			
Age (mean, SD)	51.56 (20.24)	57.07 (17.66)	<0.05
Female	32 (50.0)	74 (44.6)	0.554
Male	32 (50.0)	92 (55.4)	
White/Caucasian	41 (64.1)	60 (36.1)	<0.001
Asian	1 (1.6)	5 (3.0)	
Other	7 (10.9)	61 (36.7)	
African American	15 (23.4)	40 (24.1)	
Hispanic	1 (1.6)	67 (40.4)	<0.001
Comorbidities			
Cardiac dysfunction	14 (21.9)	28 (16.9)	0.490
Chronic kidney disease	11 (17.2)	28 (16.9)	1.000
Lung disease	15 (23.4)	27 (16.3)	0.284
Liver disease	3 (4.7)	3 (1.8)	0.443
Stroke	8 (12.5)	13 (7.8)	0.397
Immunosuppression	9 (14.1)	11 (6.6)	0.125
Cancer	15 (23.4)	14 (8.4)	<0.05
Diabetes	17 (26.6)	67 (40.4)	<0.05
BMI			
<30 (not obese)	38 (61.3)	63 (43.8)	<0.05
>30 (obese)	24 (38.7)	81 (56.2)	
Medications			
ACE inhibitor prior to admission	12 (18.8)	41 (24.7)	0.067
Angiotensin receptor blocker prior to admission	8 (12.5)	16 (9.6)	
COVID-19 clinical indicators and treatments			
Received steroids	NA	73 (44.0)	
Received remdesivir	NA	39 (23.8)	
d-Dimer (mean, SD)	NA	795.06 (1,006.82)	
MAP on sampling date (mean, SD)	NA	89.42 (12.25)	
Days from symptom onset to sampling date (mean, SD)	NA	10.50 (11.03)	
Hospitalization status			
Admitted	NA	142 (85.5)	
Not admitted	NA	24 (14.5)	
Oxygen requirement			
None	NA	48 (28.9)	
Low-flow oxygen	NA	54 (32.5)	
High-flow oxygen	NA	3 (1.8)	
Mechanical ventilation	NA	61 (36.7)	
Mortality			
Deceased	NA	25 (15.1)	
Recovered	NA	141 (84.9)	

a BMI, body mass index; ACE, angiotensin-converting enzyme; SD, standard deviation; NA, not applicable; MAP, mean arterial blood pressure in mm Hg.

b All values in the COVID+/− columns are given as *n* (%) unless otherwise specified.

c *P* values from chi-square analysis, Fisher’s exact test for categorical variables and, *t* test for continuous variables.

10.1128/msphere.00220-22.3 TABLE S1 Patient demographics and clinical characteristics by angiotensin (Ang) 1–7 quartile. Download Table S1, PDF file, 0.1 MB. Copyright © 2022 Carpenter et al. 2022 Carpenter et al. https://creativecommons.org/licenses/by/4.0/ This content is distributed under the terms of the Creative Commons Attribution 4.0 International license.

### Reduced Ang 1–7 levels associated with severe disease.

**FIG 2:**
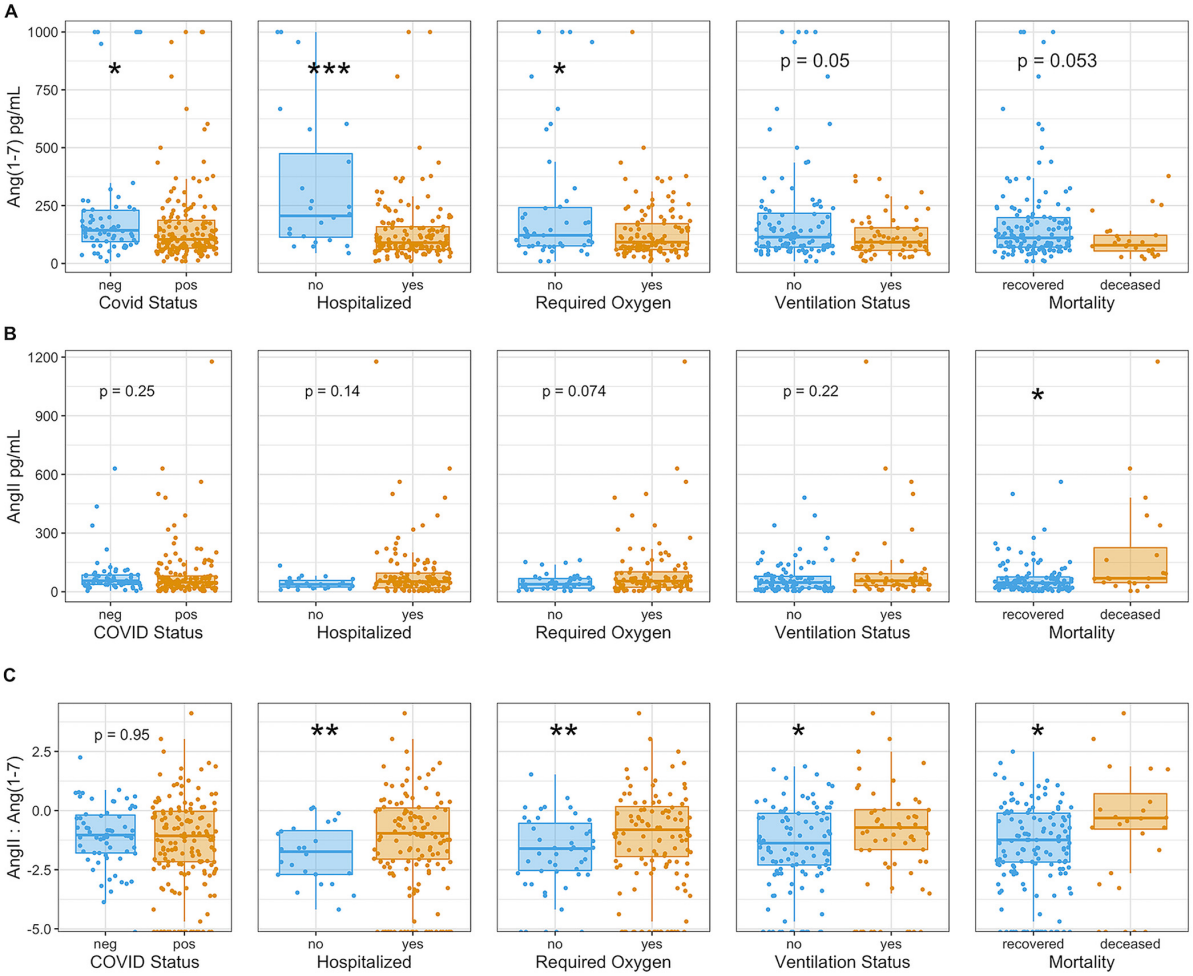
Reduced ACE2 activity evidenced by repressed Ang 1–7 and increased Ang II:Ang 1–7 ratios. (A and B) Boxplots show the distribution of Ang 1–7 and Ang II levels (pg/mL) stratified by COVID-19 status and adverse outcomes, including hospitalization, need for oxygen supplementation, ventilation, and death. Above each outcome is the unadjusted *P* value from nonparametric Wilcoxon Mann-Whitney U tests. The numbers of unique patient samples quantified are as follows: Angiotensin II (*n* = 202), Angiotensin 1–7 (*n* = 229). (C) The log-transformed ratio of Ang II to Ang 1–7 is displayed. *t* test *P* values are included above each boxplot from analyzing each outcome category separately. *, *P* < 0.05; **, *P* < 0.005; ***, *P* < 0.0005.

Ang 1–7 levels were reduced in COVID-19 patients compared to that in control subjects and decreasing levels of this peptide were correlated with disease severity (Fig. 2A). Ang 1–7 levels decreased in COVID-19 patients with increasing days from symptom onset (Fig. S1 in the supplemental material). We observed a difference in plasma Ang 1–7 levels between COVID-19 patients and controls (Wilcoxon Mann-Whitney U *P* value = 0.015). The median was 104 (interquartile range [IQR], 66 to 186) pg/mL in the COVID-19 patient group and 143 (94 to 229) pg/mL in the control group. We also observed reduced Ang 1–7 levels in patients requiring hospitalization (*P* = 0.00016), oxygen supplementation (*P* = 0.0379), and ventilation (*P* = 0.05), and among those who died of the disease (*P* = 0.053). The median in the hospitalized group was 90 (IQR, 60 to 159) pg/mL compared to 206 (113 to 474) pg/mL in the group that did not require hospitalization. The median Ang 1–7 level of those that required oxygen was 92 (IQR, 60 to 172) pg/mL compared to 122 (76 to 242) pg/mL among those who did not require oxygen. For the ventilated group, the median was 92 (IQR, 57 to 154) pg/mL versus 114 (70 to 217) pg/mL in the group that did not require ventilation. Finally, the median Ang 1–7 level of those who died of COVID-19 was 79 (IQR, 54 to 122) pg/mL versus 110 (70 to 198) pg/mL in the group that recovered.

10.1128/msphere.00220-22.1 FIG S1 Inverse relationship between Ang 1–7 and days from symptom onset in COVID-19 patients. Linear regression between Ang 1–7 and days since symptom onset in SARS-CoV-2 positive patients. Days from symptom onset were scored as per the methods of Lucas et al. (47), based on the patient’s determination or by the earliest reported symptom as recorded in the electronic medical record (*n* = 121). Download FIG S1, TIF file, 2.1 MB. Copyright © 2022 Carpenter et al. 2022 Carpenter et al. https://creativecommons.org/licenses/by/4.0/ This content is distributed under the terms of the Creative Commons Attribution 4.0 International license.

It is important to note that these measurements reflect circulating levels of Ang II and Ang 1–7, which are one-step removed from the local tissue levels, where SARS-CoV-2 directly interacts with the ACE-2 receptor. Given the extreme heterogeneity of COVID-19 in terms of patients impacted and pathophysiologic responses, any significant difference detected at the systemic level may reflect a larger difference at the tissue level, where SARS-CoV-2 inflicts damage (4, 11).

We observed an increasing trend of Ang II in more severe outcomes of COVID-19; however, mortality was the only endpoint for which the difference was statistically significant (Wilcoxon Mann-Whitney *P* = 0.0142) (Fig. 2B). The median (IQR) Ang II level in the group that died from COVID-19 was 69 (47 to 225) pg/mL, and in the group that recovered, the median (IQR) was 45 (19 to 76) pg/mL. Ang II binds to AT_1_Rs on renal juxtaglomerular cells, serving as a well-known negative feedback regulator of juxtaglomerular cell renin release and, thus, of plasma renin activity. Therefore, it might be predicted that reduced ACE2 activity in COVID-19 would have a relatively larger impact on Ang 1–7 levels than on Ang II levels, which would be expected to rapidly decline as a result of the AT_1_R short-loop negative feedback mechanism (20). It is also important to note that the ratio of Ang II:Ang 1–7 is significantly increased in more severe COVID-19 cases (Fig. 2C).

**FIG 3:**
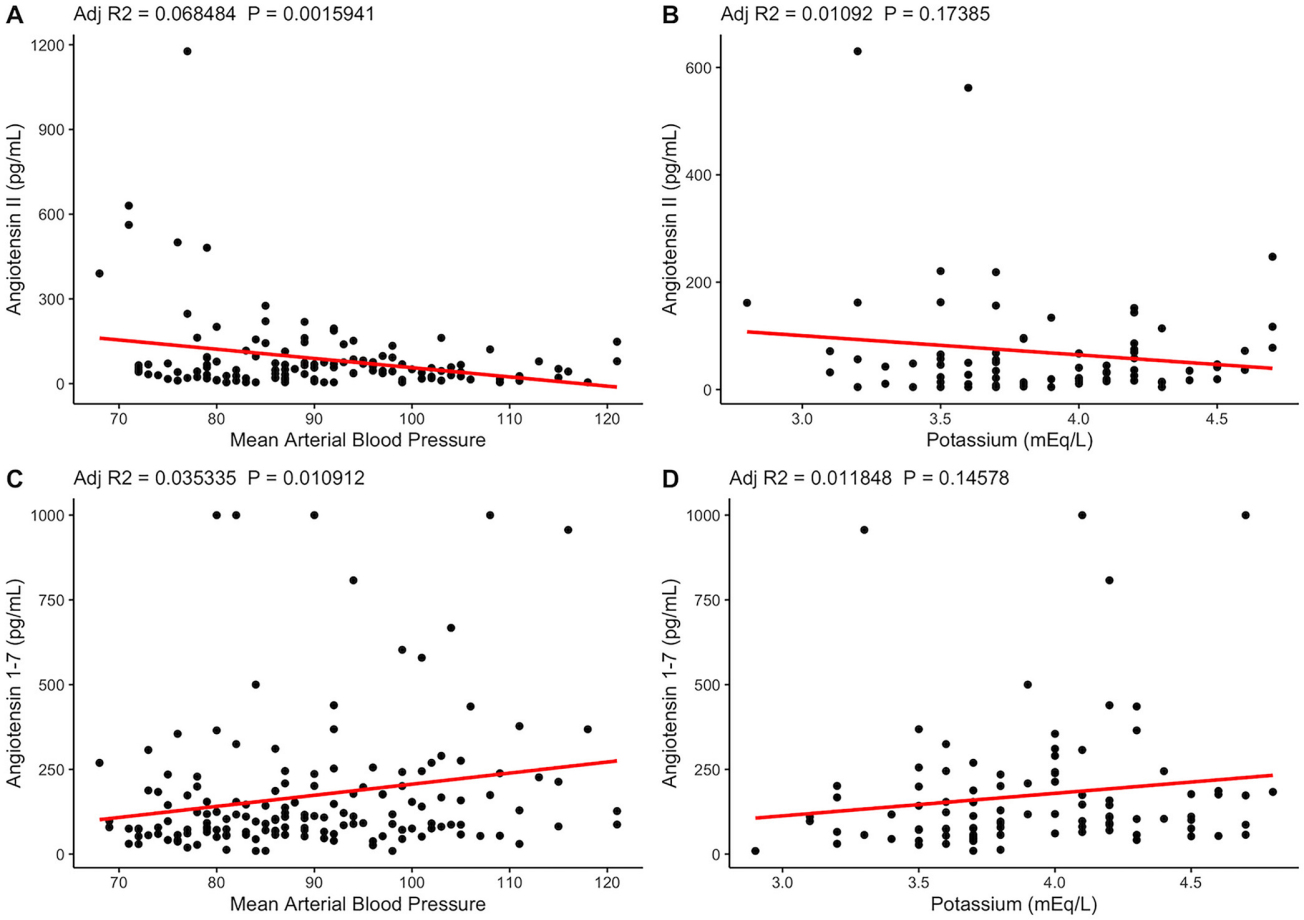
Inverse relationship of Ang II and Ang 1–7 to mean arterial blood pressures (MAP) (A and B) Linear regression between Ang II and Ang 1–7 levels and MAP averaged from all available readings taken on the day of sample collection. (C and D) Linear regression between Ang II and Ang 1–7 and potassium levels collected from the day of sample collection. The numbers of measurements available on the day of sample collection are as follows: MAP (*n* = 156 mm Hg), Potassium (mEq/L) (*n* = 98).

**FIG 4:**
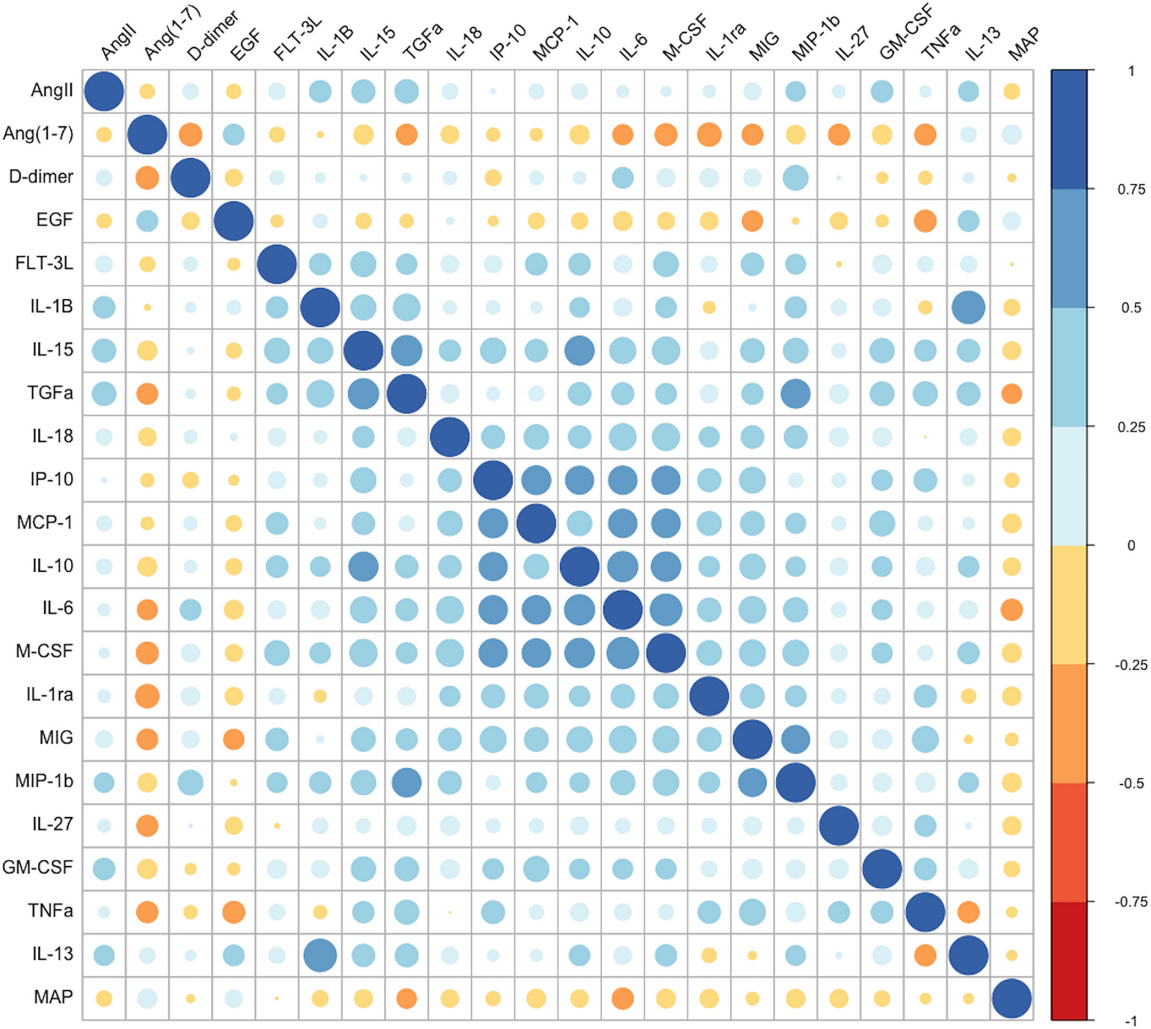
Correlation between angiotensin peptides, d-dimer, and pro-inflammatory cytokines in COVID-19. Correlation matrix depicts the Spearman’s correlation coefficient between angiotensin 1–7, angiotensin II, d-dimer, and cytokine levels on a colorimetric scale from negative correlation in red to positive correlation in blue. The cytokines displayed were selected based on significant associations with Angiotensin 1–7 levels using Kruskal-Wallis tests and analyzing each cytokine separately. The numbers of unique patient samples quantified are as follows: all cytokines (*n* = 164), angiotensin II (*n* = 202), angiotensin 1–7 (*n* = 229), d-dimer retrospectively pulled from clinical records (*n* = 54), MAP (*n* = 156).

To further explore RAS dysregulation in this cohort, we examined blood pressure and potassium, which are both impacted by Ang II levels. Ang II activates AT_1_Rs which stimulate adrenal aldosterone production (9). Aldosterone acts at the renal cortical collecting duct to promote sodium reabsorption and extracellular fluid volume expansion and increase potassium excretion (21). Thus, Ang II causes a rise in blood pressure (BP), whereas Ang 1–7 via *Mas*R opposes this mechanism, leading to natriuresis and reduction in BP (21). Interestingly, in patients with COVID-19, the opposite relationship of that anticipated between Ang II, Ang 1–7, and BP was observed (Fig. 3A and B). Ang II was negatively correlated with BP, while Ang 1–7 was positively correlated with BP, in COVID-19. This finding is consistent with an association between reduced ACE2 activity (reflected by increasing Ang II and reduced Ang 1–7) and increased inflammation leading to lower BP in more severe cases of COVID-19. Of note, BP negatively correlated with proinflammatory cytokines (Fig. 4). However, it is uncertain whether this was a direct cause of inflammation or was confounded by the high rates of sedation and positive pressure ventilation in more severe manifestations of disease. We did not observe a significant association between Ang peptides and potassium in this cohort. However, we were unable to control for potassium repletion and the impact of acute kidney injury, which occurred in over 30% of patients, on circulating potassium levels (Fig. 3C and D).

### Ang 1–7 predicts need for hospitalization and oxygen supplementation in COVID-19.

We fit logistic regression models for hospitalization, oxygen supplementation, ventilation, and mortality separately with predictors, including Ang II, Ang 1–7, age, sex, race, BMI, ACEi/ARB use, and the presence of any comorbidity, categorized as yes/no (Table 2). In univariable analysis, Ang 1–7, age, and the presence of any comorbidity were associated with need for hospitalization. All of these, in addition to Ang II and ACE inhibitor (ACEi)/AT_1_R blocker (ARB) use, were associated with need for oxygen supplementation. Table 2 also illustrates multivariable logistic regression analyses using significant variables selected during univariable analyses. Every 10-pg/mL unit increase in plasma Ang 1–7 levels was associated with a 3% reduction in odds of hospitalization (adjusted odds ratio [AOR] 0.97, confidence interval [CI] 0.95 to 0.99) and a 3% reduction in odds of requiring oxygen supplementation (AOR 0.97, CI 0.95 to 0.99) and/or ventilation (AOR 0.97, CI 0.94 to 0.99). No significant association was noted on multivariable analysis between Ang 1–7 levels and mortality (Table 2). In contrast, as we might expect, every 10-pg/mL increase in Ang II levels was associated with a 4% increase in odds of mortality on multivariable analysis (AOR 1.04, CI 1.01 to 1.08).

**TABLE 2:** Predictors of hospitalization and severe respiratory illness in COVID-19^*a*^

Characteristic	Unadjusted		Adjusted	
	OR (95% CI)^*b*^	*P* value^*b*^	AOR (95% CI)	*P* value
Hospitalization				
Ang II (per 10 pg/mL)	1.10 (1.01–1.24)	0.0708	1.08 (0.99–1.24)	0.195
Ang 1–7 (per 10 pg/mL)	0.96 (0.94–0.98)	0.000274^*b*^	0.97 (0.95–0.99)	0.00989^*b*^
Age (per 10 yrs)	1.74 (1.32–2.37)	0.000164^*b*^	1.52 (1.06–2.27)	0.0309^*b*^
Sex (male)	1.40 (0.74–2.68)	0.302	-	-
Race (ref = White/Caucasian)				
African American	0.29 (0.07–0.98)	0.0544	0.15 (0.02–0.71)	0.0234^*b*^
Asian	0.12 (0.01–0.98)	0.0333	0.30 (0.02–3.85)	0.337
Other	0.36 (0.10–1.16)	0.105	0.73 (0.15–3.04)	0.666
Ethnicity (Hispanic)	0.94 (0.39–2.32)	0.888	-	-
BMI (>30, obese)	1.26 (0.41–3.89)	0.678	-	-
ACE inhibitor	4.38 (1.20–28.29)	0.0538	2.76 (0.53–22.18)	0.268
ARB	1.57 (0.40–10.52)	0.569	0.74 (0.12–6.38)	0.754
Any Comorbidity	4.76 (1.91–13.03)	0.00124^*b*^	2.33 (0.74–7.74)	0.153
Oxygen requirement				
Ang II (per 10 pg/mL)	1.07 (1.02–1.16)	0.0327^*b*^	1.07 (1.01–1.17)	0.0903
Ang 1–7 (per 10 pg/mL)	0.97 (0.95–0.99)	0.00332^*b*^	0.97 (0.95–0.99)	0.0144^*b*^
Age (per 10 yrs)	1.03 (1.01–1.05)	0.0066^*b*^	1.19 (0.93–1.54)	0.172
Sex (male)	0.95 (0.48–1.87)	0.891	-	-
Race (ref = White/Caucasian)				
African American	0.78 (0.32–1.92)	0.582	-	-
Asian	0.50 (0.08–4.08)	0.470	-	-
Other	0.74 (0.33–1.63)	0.453	-	-
Ethnicity (Hispanic)	0.73 (0.37–1.44)	0.360	-	-
BMI (>30, obese)	1.82 (0.82–4.08)	0.141	-	-
ACE inhibitor	2.76 (1.13–7.83)	0.0372^*b*^	1.73 (0.57–6.06)	0.354
ARB	0.61 (0.21–1.83)	0.361	0.33 (0.09–1.19)	0.0860
Any Comorbidity	2.12 (1.07–4.22)	0.0310^*b*^	1.55 (0.65–3.67)	0.317
Ventilated				
Ang II (per 10 pg/mL)	1.03 (1.00–1.06)	0.0869	1.00 (1.00–1.01)	0.163
Ang 1–7 (per 10 pg/mL)	0.97 (0.94–0.99)	0.021^*b*^	0.97 (0.94–0.99)	0.0455^*b*^
Age (per 10 yrs)	1.07 (0.89–1.28)	0.484	-	-
Sex (male)	1.40 (0.74–2.68)	0.302	-	-
Race (ref = White/Caucasian)				
African American	0.66 (0.27–1.54)	0.341	-	-
Asian	1.15 (0.14–7.47)	0.882	-	-
Other	1.28 (0.62–2.68)	0.503	-	-
Ethnicity (Hispanic)	1.29 (0.68–2.45)	0.435	-	-
BMI (>30 obese)	1.47 (0.75–2.89)	0.263	-	-
ACE inhibitor	1.78 (0.86–3.70)	0.121	-	-
ARB	0.43 (0.09–1.44)	0.210	-	-
Any comorbidity	0.89 (0.47–1.70)	0.713	-	-
Mortality				
Ang II (per 10 pg/mL)	1.05 (1.02–1.09)	0.00413^*b*^	1.04 (1.01–1.08)	0.0302^*b*^
Ang 1–7 (per 10 pg/mL)	0.96 (0.91–1.00)	0.106	1.00 (0.99–1.00)	-
Age (per 10 yrs)	1.73 (1.30–2.38)	0.000319^*b*^	1.65 (1.19–2.41)	0.00497^*b*^
Sex (male)	1.25 (0.53–3.05)	0.618	-	-
Race (ref = White/Caucasian)				
African American			-	-
Asian			-	-
Other			-	-
Ethnicity (Hispanic)	0.65 (0.25–1.57)	0.358	-	-
BMI (>30, obese)	0.63 (0.25–1.57)	0.318	-	-
ACE inhibitor	1.65 (0.61–4.21)	0.307	-	-
ARB	1.57 (0.33–5.64)	0.523	-	-
Any comorbidity	2.96 (1.13–9.31)	0.0398^*b*^	1.31 (0.41–4.76)	0.657

a OR, odds ratio; 95% CI, 95% confidence interval; AOR, adjusted odds ratio; BMI, body mass index; ACE, angiotensin-converting enzyme; ARB, angiotensin receptor blocker.

b OR, 95% CI, and *P* values obtained from univariable and multivariable regression models. Independent variables identified in univariable models to be associated with adverse outcomes (*P* < 0.10) were included in a multivariable binomial logistic regression.

Since many of the patients included in this analysis contracted COVID-19 prior to the establishment of standard treatments for the disease, less than half of these patients received immunomodulatory and antiviral therapy. Of those who did, the majority received a single dose prior to blood sample collection. Regarding the influence of immunomodulatory therapy on RAS peptide levels, median Ang 1–7 concentrations in patients treated with corticosteroids (compared with those in patients not treated with corticosteroids) were 110 (IQR, 72 to 187) and 91 (60 to 178) pg/mL, respectively (*P* = 0.534). Median Ang II concentrations in those treated with corticosteroids (compared to no corticosteroids) were 58 (IQR, 25 to 115) and 44 (18 to 69) pg/mL, respectively (*P* = 0.072). Antiviral treatment did not have a statistically significant correlation with RAS metabolite concentrations (data not shown).

### Ang 1–7, inflammation, and coagulation.

Ang 1–7 has anti-inflammatory and anti-thrombotic effect via activation of the *Mas*R (22, 23). This peptide inhibits pro-inflammatory cytokines interleukin (IL)-6, tumor necrosis factor (TNF)-α, IL-1β, and monocyte chemoattractant protein (MCP)-1 through the NF-κB, Jun N-terminal protein kinase (JNK) and extracellular signal-regulated kinase (ERK) 1/2 pathways (24, 25). To investigate the impact of Ang 1–7 on the inflammatory response to SARS-CoV-2, we grouped patients into Ang 1–7 quartiles in which a significantly greater proportion of the lowest versus the highest groupings required hospitalization (Table 3). We then evaluated each cytokine, growth factor, and d-dimer individually for significant differences across Ang 1–7 quartiles (data not shown). Factors identified as having significant associations with Ang 1–7 were incorporated into a correlation matrix to determine the strength and direction of each association (Fig. 4). As expected, Ang II was positively correlated with most pro-inflammatory cytokines, while Ang 1–7 was negatively correlated with both cytokines and d-dimer levels. A medium-strength association was detected between d-dimer, pro-inflammatory cytokines IL-6 and TNF-α, and CXCL9 and macrophage colony-stimulating factor (M-CSF), which are involved in monocyte signaling. This finding supports our hypothesis that increasing Ang 1–7 levels are protective in COVID-19 via the inhibition of thrombosis and inflammation (11, 26). Interestingly, anti-inflammatory cytokine IL-10 was also negatively associated with Ang 1–7. On further exploration, this increase in IL-10 in the lower Ang 1–7 groups was associated with immune activation of pro-inflammatory cytokines, and could be a compensatory response (Fig. S2) (27).

**TABLE 3:** Patient demographics and characteristics by Ang 1–7 quartile^*a*^

Characteristic	Ang 1–7 quartile				*P* value^*b*^
	1st (*n* = 42)	2nd (*n* = 41)	3rd (*n* = 41)	4th (*n* = 41)	
Demographics					
Age (mean, SD)	62.07 (16.64)	56.63 (18.92)	60.05 (16.79)	49.10 (16.06)	0.004
Sex, *n* (%)					0.471
Female	18 (42.9)	15 (36.6)	19 (46.3)	22 (53.7)	
Male	24 (57.1)	26 (63.4)	22 (53.7)	19 (46.3)	
Race, *n* (%)					0.055
African American	13 (31.0)	7 (17.1)	12 (29.3)	7 (17.1)	
Asian	1 (2.4)	0 (0.0)	0 (0.0)	4 (9.8)	
Other	12 (28.6)	14 (34.1)	16 (39.0)	19 (46.3)	
White/Caucasian	16 (38.1)	20 (48.8)	13 (31.7)	11 (26.8)	
Ethnicity, *n* (%)					0.606
Hispanic	15 (35.7)	17 (41.5)	15 (36.6)	20 (48.8)	
Comorbidities, *n* (%)					
Cardiac dysfunction	12 (28.6)	7 (17.1)	4 (9.8)	4 (9.8)	0.066
Chronic kidney disease	7 (16.7)	7 (17.1)	6 (14.6)	8 (19.5)	0.95
Lung disease	7 (16.7)	8 (19.5)	8 (19.5)	4 (9.8)	0.592
Liver disease	0 (0.0)	1 (2.4)	2 (4.9)	0 (0.0)	0.287
Stroke	3 (7.1)	4 (9.8)	3 (7.3)	2 (4.9)	0.867
Immunosuppression	3 (7.1)	2 (4.9)	4 (9.8)	2 (4.9)	0.785
Cancer	4 (9.5)	4 (9.8)	4 (9.8)	2 (4.9)	0.822
Diabetes	19 (45.2)	17 (41.5)	14 (34.1)	17 (41.5)	0.775
BMI	15 (38.5)	13 (37.1)	18 (51.4)	16 (47.1)	0.562
<30 (not obese)	24 (61.5)	22 (62.9)	17 (48.6)	18 (52.9)	
>30 (obese)	12 (28.6)	7 (17.1)	4 (9.8)	4 (9.8)	
Medications, *n* (%)					
ACEi prior to admission	14 (33.3)	12 (29.3)	8 (19.5)	7 (17.1)	0.176
ARB prior to admission	4 (9.5)	6 (14.6)	4 (9.8)	1 (2.4)	0.176
COVID-19 clinical indicators					
Symptom onset to sampling date (days)	11.65 (8.70)	14.21 (17.73)	10.55 (8.06)	5.74 (4.32)	0.024
MAP (SD)	86.28 (11.13)	87.59 (12.95)	90.31 (10.69)	93.39 (13.23)	0.047
d-Dimer (mean, SD)	1,042.64 (897.64)	603.33 (282.71)	1,001.50 (1,553.44)	422.67 (479.44)	0.315
Hospitalization status, *n* (%)					0.002
Admitted	41 (97.6)	36 (87.8)	36 (87.8)	28 (68.3)	
Not admitted	1 (2.4)	5 (12.2)	5 (12.2)	13 (31.7)	
Oxygen requirement, *n* (%)					0.299
None	7 (16.7)	13 (31.7)	12 (29.3)	15 (36.6)	
Low-flow oxygen	17 (40.5)	11 (26.8)	12 (29.3)	14 (34.1)	
High-flow oxygen	0 (0.0)	1 (2.4)	0 (0.0)	2 (4.9)	
Mechanical ventilation	18 (42.9)	16 (39.0)	17 (41.5)	10 (24.4)	
Mortality, *n* (%)					0.290
Deceased	9 (21.4)	8 (19.5)	4 (9.8)	4 (9.8)	
Recovered	33 (78.6)	33 (80.5)	37 (90.2)	37 (90.2)	

a Ang, angiotensin; SD, standard deviation; BMI, body mass index; ACEi, angiotensin-converting enzyme inhibitor; ARB, angiotensin receptor blocker; MAP, mean arterial blood pressure in mm Hg.

b *P* values from chi-square analysis, Fisher’s exact test for categorical variables, and *t* test for continuous variables.

10.1128/msphere.00220-22.2 FIG S2 Reactive increase in interleukin (IL)-10 correlates with proinflammatory cytokines. Proinflammatory cytokines, tumor necrosis factor α (TNF-α), monocyte chemoattractant protein 1 (MCP-1), IL-6, and IL-1B, along with IL-10, were log-transformed and then input into linear regression (*n* = 164). Download FIG S2, TIF file, 2.6 MB. Copyright © 2022 Carpenter et al. 2022 Carpenter et al. https://creativecommons.org/licenses/by/4.0/ This content is distributed under the terms of the Creative Commons Attribution 4.0 International license.

## DISCUSSION

Here, we have shown that reduced Ang 1–7 is associated with hospitalization, oxygen supplementation, and ventilation in COVID-19, particularly when adjusted for age, race, use of ACEi/ARBs, and comorbidity status. The most significant difference in Ang 1–7 levels was detected between relatively asymptomatic cases and patients requiring some degree of medical intervention. The SARS-CoV-2-negative cohort, being less sick than the COVID-positive cohort, does not allow this association between decreased Ang 1–7 plasma levels and more severe COVID-19 to be attributed to COVID-19 specifically as opposed to critical illness more generally. However, this does not diminish the importance of the results, or the opportunity presented for potential COVID-19 therapies.

Our findings here are in contradistinction to those of a recent comprehensive meta-analysis which found overactivation, rather than depression, of the protective arm of the RAS in COVID-19 (8). This meta-analysis does note significant differences among the results of the included studies, but reports that on average, Ang 1–7 levels are approximately 10 times higher in COVID-19 patients than in the controls (8). Several explanations could account for the discrepancy in these findings. First, this meta-analysis combined a range of studies which employed different sample collection and processing methods. The largest included study employed equilibrium analysis, which does not utilize protease inhibitors and reflects the ongoing activity of RAS proteases (28, 29). Given that ACE2 is cleaved from the cell surface and that circulating levels are increased in COVID-19, equilibrium analysis does not provide a reliable indication of RAS metabolite levels in the body tissues because Ang 1–7 production continues after serum collection. Gheware et al. (30) reported increased levels of ACE2 protein expression in lung tissues of patients who died of COVID-19 based on gross estimation using immunohistochemistry. However, it is unclear whether ACE2 in these lung tissues is intracellular or extracellular, and the activity of the enzyme was not explored. It is possible that ACE2 protein expression is increased in COVID-19 to compensate for increased receptor internalization and decreased activity. Other studies allowed blood samples to clot at room temperature and reported RAS peptide levels at surprisingly low concentrations, suggesting that ongoing peptide metabolism confounded the findings (31–33). Second, most studies investigating RAS dysregulation in COVID-19 are small and underpowered, with the largest study including only 126 patients, of which only 32 were severely ill (29). Other studies included few to no severely ill COVID-19 patients (28, 31–33).

Our study agrees with the findings of Henry et al. (34), who found Ang 1–7 levels to be significantly lower in patients with COVID-19 compared to those in controls, and in those admitted to the ICU versus those who did not require intensive care. Similarly, Wu et al. (35) and Liu et al. (36) reported significant elevations in Ang II levels in critically ill COVID-19 cases compared to those in controls/mild cases.

The primary strength of this study is the inclusion of a large COVID-19 patient cohort with disease phenotypes ranging from asymptomatic to critical illness. This large and diverse cohort has allowed the identification of RAS dysregulation in COVID-19 that was consistent across outcomes and significant upon adjusted multivariable analysis. Given the low endogenous concentration of the RAS peptides and the considerable heterogeneity seen in COVID-19 patients, a large patient cohort with the power to detect small changes in the RAS is critical to successful determination of the impact of SARS-CoV-2 on this tightly regulated system (37, 38). Given the sample size of 166 COVID-positive patients, we were also able to control for the impacts of race, age, comorbidities, and long-term ACEi/ARB use in multivariate analysis. Dexamethasone, which is now the standard of care in severe COVID-19, upregulates ACE and ACE2 and decreases morbidity and mortality (39). To ensure that immunomodulatory therapy did not confound the current analysis, we compared initial RAS metabolite levels in those who received treatment versus those who did not and found no significant difference. Further studies at various time points before, during, and after treatment are needed to determine whether dexamethasone provides therapeutic benefit via upregulation of ACE2 and subsequent alterations in RAS metabolite levels.

Another strength is the of pairing Ang peptide with cytokine and d-dimer levels, which demonstrated significant associations, consistent with prior research regarding the role of the ACE2/Ang 1–7/MasR pathway in modulating inflammation and coagulation (22, 25). To our knowledge, this is the first study to examine the association of RAS peptides and cytokine/d-dimer levels in COVID-19. Association of reduced Ang 1–7 with increased inflammation/thrombosis strengthens the level of evidence that disruptions in this counter-regulatory pathway are involved in the pathogenesis of COVID-19 (18).

The mechanism(s) by which Ang 1–7 protects against severe disease is/are unknown, although some clues can be taken from the literature and from correlations in this cohort with inflammatory markers and d-dimer levels. Many studies have shown that the RAS, composed of the ACE/Ang II/AT_1_R axis and the counterregulatory ACE2/Ang 1–7/*Mas*R pathway, plays a relevant role in the pathogenesis of inflammatory diseases (25). Ang II is known to activate signaling pathways related to tissue injury, inflammation, and fibrosis, including activation of the transcription factor NF-κB, recruitment of inflammatory cells, adhesion of monocytes and neutrophils to endothelial and mesangial cells, and synthesis and release of cytokines and chemokines, including IL-1β (40). Evidence suggests that Ang 1–7 opposes these actions, as the heptapeptide has been shown to downregulate mRNA levels of pro-inflammatory cytokines IL-6 and TNF-α, negatively modulate leukocyte migration, and decrease the frequency of M1 inflammatory macrophage phenotypes (40, 41). SARS-CoV-2 appears to downregulate the expression of ACE-2 on peripheral blood monocytes which show an activated phenotype in COVID-19 evidenced by morphology and IL-6, IL-10, and TNF-α production (31, 42, 43). Monocyte activation appears to associate with disease severity, and macrophage accumulation has been noted in COVID-19 patients on autopsy along with diffuse alveolar damage, pulmonary edema, fibrin deposition in the alveolar space, and diffuse microvascular thrombi (42, 44). It is interesting to note that Ang II was positively correlated with IL-1β and granulocyte-macrophage colony-stimulating factor (GM-CSF) in this cohort, while Ang 1–7 was negatively correlated with IL-6, TNF-α and M-CSF (Fig. 4). While further research is needed to define the mechanism by which RAS dysfunction impacts the course of COVID-19, it is possible that reduced Ang 1–7 levels favor a pro-inflammatory activation of macrophages in severe disease. Further investigation should also determine whether RAS dysfunction is related to the pulmonary fibrosis and long-term sequelae of severe COVID-19 (45).

Ang 1–7 levels are also relevant to the risk of thrombosis in COVID-19 through a relatively direct mechanism. Ang 1–7 normally acts on the *Mas* and AT_2_ receptors to increase the production of nitric oxide and prostacyclin, which in turn contribute to vasodilation, reduced platelet spreading, and collagen activation (22). Loss of this protective activity in COVID-19 likely contributes to the diffuse microvascular thrombi seen in many patients. This is supported by our finding that Ang 1–7 is negatively correlated with d-dimer levels in COVID-19 patients, suggesting reduced thrombi formation and break-down among patients with higher Ang 1–7 levels (Fig. 4).

### Limitations.

While the discarded sample design of this study was necessary to achieve this large cohort, it does introduce a limitation in that it is not possible to prevent 100% of Ang II/Ang 1–7 breakdown by proteases. To address this limitation, sample storage and processing were standardized, multiple freeze-thaw cycles were excluded, and EDTA was used, which is consistent with other studies and has previously been shown to stabilize Ang II and Ang 1–7 in plasma (31, 35, 37, 38). Additionally, according to the advice put forth by Chappell et al. (46), we utilized plasma instead of serum and stored the samples at −80°C prior to analysis. Finally, measuring circulating Ang II/Ang 1–7 levels does not fully capture the impact of SARS-CoV-2 at the tissue level. However, having detected consistent Ang 1–7 repression in this cohort, we expect that the magnitude of this repression at the tissue level may be larger than what is reported here.

## MATERIALS AND METHODS

### Study design.

Discarded human plasma samples from COVID-19 positive and negative patients at the University of Virginia (UVA) Medical Center were collected for cytokine, angiotensin peptide, and growth factor analyses. The collection of biological specimens and de-identified patient information (no consent required) was approved by the UVA Institutional Review Board (IRB-HSR no. 22231 and 200110).

### Human samples.

Blood samples from 230 patients tested for SARS-CoV-2 by PCR between April and October 2020 were found using the UVA Medical Center’s electronic database. In total, 166 of the 230 patients included in this study were SARS-CoV-2-positive and 64 were SARS-CoV-2-negative. For those patients with COVID-19, the earliest blood samples taken during emergency department visit or hospitalization at UVA were used for this analysis. The SARS-CoV-2-negative group was randomly selected from healthy controls who visited the UVA for an outpatient appointment requiring SARS-CoV-2 screening. Blood was collected into EDTA-containing vacutainers by a trained hospital phlebotomist. Blood was centrifuged at 1,300 × *g* for 10 min, and after completion of biochemical testing, as ordered by the clinician, the remaining plasma was stored at 4°C for 48 h before it was deemed “discarded” and released to the research laboratory. Plasma samples were aliquoted and stored at −80°C until immediately prior to testing.

### Patient descriptors/clinical course.

Demographics (age, gender, race), comorbidities, medication use, hospitalization status, lab results, and other clinical information were obtained by an honest broker from the electronic medical record (EMR) (Table 1, Table S1). Confidentiality was maintained by assigning each patient a unique identifier. Severity of COVID-19 illness was assessed through review of the EMR in several ways: first by inpatient admission versus outpatient care, second by the use of supplemental oxygen (none versus any supplemental oxygen, and supplemental oxygen delineated as low-flow nasal canula versus mechanical ventilation or high-flow oxygen [>15 L per min]), and finally by mortality. Days from symptom onset were scored as per the methods of Lucas et al. (47), based on the patient’s determination or on the earliest reported symptom from the patient as recorded in the electronic medical record. All mean arterial blood pressure measurements from the day of sample collection were pulled from the electronic medical record and averaged prior to inclusion in all analyses.

### Quantification of Ang II.

Ang II was quantified in undiluted plasma using the Angiotensin II ELISA kit (ALPCO, cat no. 74-ANGHU-E01) according to the manufacturer’s instructions. Ang II measurements from 28 of the 230 aliquoted samples were excluded from the final analysis due to the samples undergoing multiple freeze-thaw cycles. Standard curves were prepared for each 96-well plate. The minimum and maximum detectable concentrations of Ang II were 4.6 and 10,000 pg/mL, respectively.

### Quantification of Ang 1–7.

Ang 1–7 was quantified in undiluted plasma using the Angiotensin 1–7 ELISA kit (Novus Biologicals, cat no. NBP2-69078). Manufacturer instructions were followed using 35 μL of sample per well. Ang 1–7 measurements from 1 of the 230 aliquoted samples were excluded from the final analysis due to insufficient sample volume. Standard curves were prepared for each 96-well plate. The minimum and maximum detectable concentrations of Ang 1–7 were 9.38 and 1,000 pg/mL, respectively.

### Cytokine quantification.

Cytokine concentrations in plasma were measured using the MILLIPLEX MAP Human Cytokine/Chemokine/Growth Factor Panel A (48 Plex) (Millipore Sigma, St. Louis MO, cat no. HCYTA-60K-PX48) by the Flow Cytometry Facility of UVA. The cytokines detected were sCD40L, epidermal growth factor (EGF), eotaxin, fibroblast growth factor 2, Flt-3 ligand, fractalkine, granulocyte colony-stimulating factor, GM-CSF, GROα, IFN-α2, IFN-γ, IL-1α, IL-1β, IL-1 receptor antagonist, IL-2, IL-3, IL-4, IL-5, IL-6, IL-7, IL-8, IL-9, IL-10, IL-12 (p40), IL-12 (p70), IL-13, IL-15, IL-17A, IL-17E/IL-25, IL-17F, IL-18, IL-22, IL-27, IP-10, MCP-1, MCP-3, M-CSF, macrophage-derived chemokine (MDC/CCL22), MIG, MIP-1α, MIP-1β, platelet-derived growth factor (PDGF)-AA, PDGF-AB/BB, transforming growth factor α, TNF-α, TNF-β, and vascular endothelial growth factor A. (RANTES was excluded).

### D-dimer measurement.

D-dimer levels determined by the clinical laboratory at UVA on the same day as samples were collected for this study were retrospectively pulled from the electronic medical record by an honest broker.

### Statistical methods.

All statistical comparisons and graphs were made using R version 4.0.3. Cases and controls were compared with respect to Ang II and Ang 1–7 using nonparametric Mann-Whitney U tests. Key outcome indicators such as hospitalization, oxygen supplementation, ventilation, and mortality were categorized as yes/no and also compared with respect to Ang II and Ang 1–7 using nonparametric Mann-Whitney U tests. We estimated odds ratios (ORs) for the association of Ang peptides (and other independent variables) with adverse outcomes of COVID-19 using univariable logistic regression (Table 2). Independent variables identified in univariable models (*P* < 0.10) were included in multivariable logistic regression. A *P* value of <0.05 was considered statistically significant (Table 2). Patients were classified into 4 quartiles based on the cumulative distribution of Ang 1–7 levels (Table 3). Associations between individual inflammatory/coagulation markers and Ang 1–7 quartiles were detected using Kruskal-Wallis tests, and a *P* value of <0.05 was considered statistically significant (data not shown). Significant associations were further analyzed using a Spearman’s rank correlation to measure the degree of the association between inflammatory/coagulation markers and angiotensin peptides inputted as individual continuous variables. Results are summarized as showing a weak (0 to 0.25), medium (0.25 to 0.5), or strong (>0.5) association (Fig. 4).

### Data availability.

This analysis has not made use of any mandated data sets and supporting data are included within the main article and the supplementary files. Any additional data, code, materials, and associated protocols will be made available to qualified users through the UVA server upon request.
