# Supplementary material for: Repressed Ang 1–7 in COVID-19 Is Inversely Associated with Inflammation and Coagulation
Source: mSphere. 2022 Aug 1;7(4):e00220-22. doi: 10.1128/msphere.00220-22 (PMC9429950; doi:10.1128/msphere.00220-22)
Supplement: TABLE S1 [file msphere.00220-22-s0001.pdf]

Supplemental Table I | COVID-19 Patient Demographics and Clinical Characteristics by Outcome				
Characteristic	No. (%)			
	Hospitalized (n= 142)	Required Oxygen (n= 118)	Ventilated (n=61)	Deceased (n=25)
**Demographics**				
Age (mean, SD)	59.34 (16.72)	59.49 (16.04)	58.33 (14.27)	69.48 (15.74)
Sex				
Female	64 (45.1)	53 (44.9)	24 (39.3)	10 (40.0)
Male	78 (54.9)	65 (55.1)	37 (60.7)	15 (60.0)
Race				
African American	32 (22.5)	28 (23.7)	11 (18.0)	5 (20.0)
Asian	3 (2.1)	3 (2.5)	2 (3.3)	0 (0.0)
Other	51 (35.9)	42 (35.6)	26 (42.6)	6 (24.0)
White or Caucasian	56 (39.4)	45 (38.1)	22 (36.1)	14 (56.0)
Ethnicity				
Hispanic	57 (40.1)	45 (38.1)	27 (44.3)	8 (32.0)
**Comorbidities**				
Cardiac dysfunction	27 (19.0)	21 (17.8)	8 (13.1)	8 (32.0)
Chronic Kidney Disease	28 (19.7)	22 (18.6)	6 (9.8)	8 (32.0)
Lung Disease	26 (18.3)	20 (16.9)	7 (11.5)	5 (20.0)
Liver Disease	3 (2.1)	2 (1.7)	0 (0.0)	0 (0.0)
Stroke	12 (8.5)	11 (9.3)	3 (4.9)	5 (20.00)
Immunosuppression	11 (7.7)	9 (7.6)	7 (11.5)	2 (8.0)
Cancer	13 (9.2)	10 (8.5)	6 (9.8)	3 (12.0)
Diabetes	62 (43.7)	54 (45.8)	24 (39.3)	13 (52.0)
Body Mass Index				
<30 (not obese)	56 (43.1)	45 (40.5)	23 (37.7)	12 (54.5)
>30 (obese)	74 (56.9)	66 (59.5)	38 (62.3)	10 (45.5)
**Medications**				
ACE inhibitor, prior to admission	39 (27.5)	35 (29.7)	20 (32.8)	8 (32.0)
ARB, prior to admission	14 (9.9)	9 (7.6)	3 (4.9)	3 (12.0)
**COVID-19 Clinical Indicators/Treatments**				
Received Steroids	72 (50.7)	67 (56.8)	41 (62.1)	12 (48.0)
Received Remdesivir	38 (27.1)	39 (33.3)	17 (27.9)	5 (20.8)
D-dimer (Mean, SD)	795.06 (1006.82)	776.94 (1030.79)	1043.61 (1286.46)	897.00 (838.89)
MAP (Day of Sample) (Mean, SD)	88.01 (11.67)	87.07 (11.32)	83.59 (10.16)	82.96 (11.25)
Days from Symptom Onset to Sample (Mean, SD)	11.57 (11.57)	11.35 (10.44)	14.83 (13.00)	14.50 (14.58)
