# Supplementary figures and images for: Repressed Ang 1–7 in COVID-19 Is Inversely Associated with Inflammation and Coagulation
Source: mSphere. 2022 Aug 1;7(4):e00220-22. doi: 10.1128/msphere.00220-22 (PMC9429950; doi:10.1128/msphere.00220-22)

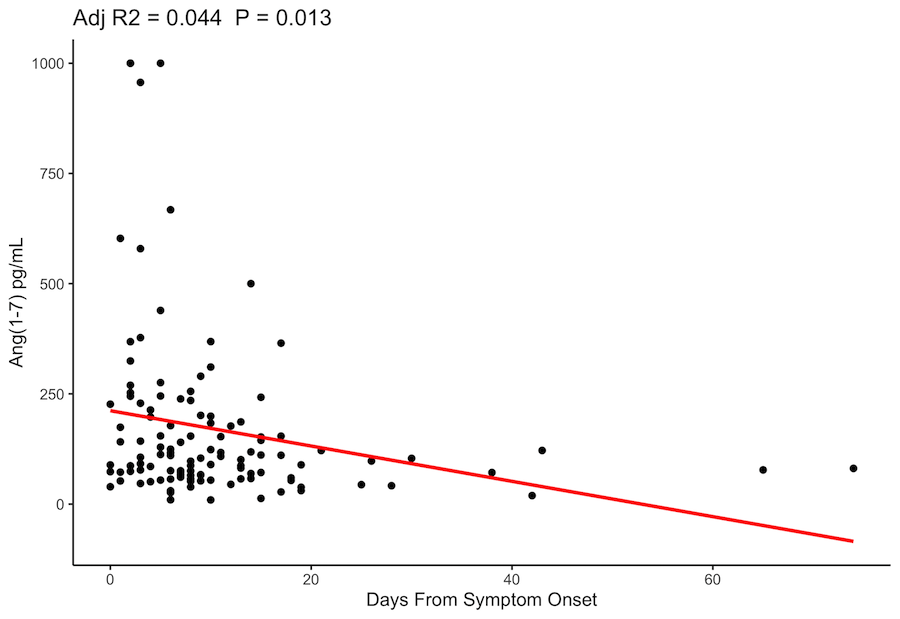

Supplement: FIG S1 [file msphere.00220-22-s0002.tif]

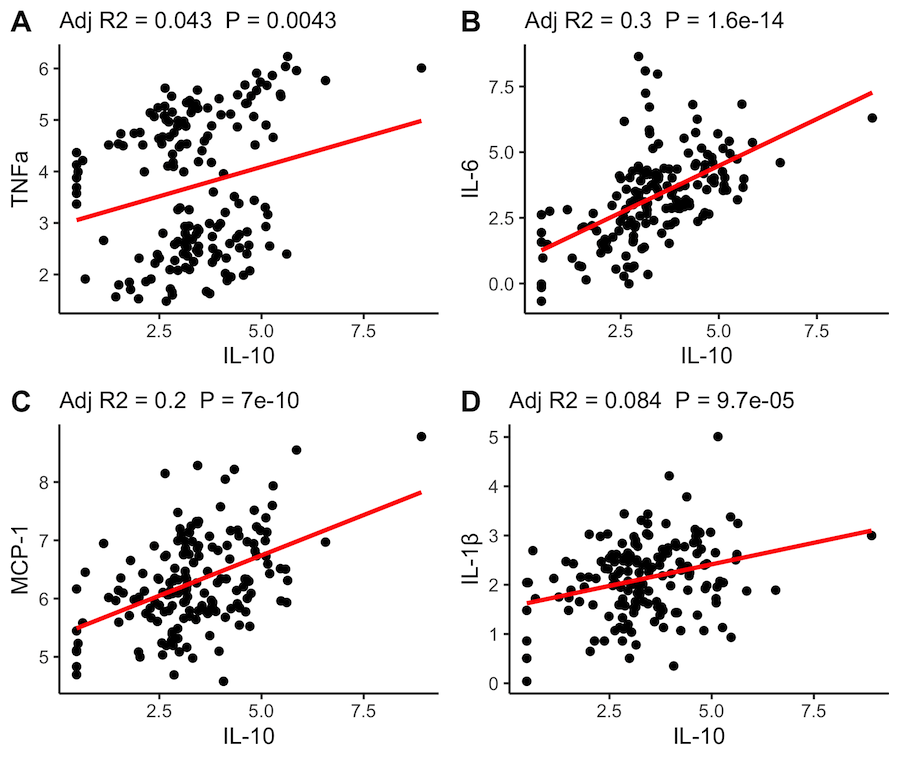

Supplement: FIG S2 [file msphere.00220-22-s0003.tif]
